# Socio-economic inequalities in the incidence of four common cancers: a population-based registry study

**DOI:** 10.1016/j.puhe.2017.10.005

**Published:** 2018-01

**Authors:** E.J. Tweed, G.M. Allardice, P. McLoone, D.S. Morrison

**Affiliations:** a MRC/CSO Social and Public Health Sciences Unit, University of Glasgow, 200 Renfield Street, Glasgow, G2 3QB, UK; b Directorate of Public Health, NHS Greater Glasgow and Clyde, West House, Gartnavel Royal Hospital, 1055 Great Western Road, Glasgow, G12 0XH, UK; c Department of Public Health, University of Glasgow, 1 Lilybank Gardens, Glasgow, G12 8RZ, UK

**Keywords:** Socio-economic circumstances, Health inequalities, Cancer incidence, Cancer registry

## Abstract

**Objectives:**

To investigate the relationship between socio-economic circumstances and cancer incidence in Scotland in recent years.

**Study design:**

Population-based study using cancer registry data.

**Methods:**

Data on incident cases of colorectal, lung, female breast, and prostate cancer diagnosed between 2001 and 2012 were obtained from a population-based cancer registry covering a population of approximately 2.5 million people in the West of Scotland. Socio-economic circumstances were assessed based on postcode of residence at diagnosis, using the Scottish Index of Multiple Deprivation (SIMD). For each cancer, crude and age-standardised incidence rates were calculated by quintile of SIMD score, and the number of excess cases associated with socio-economic deprivation was estimated.

**Results:**

93,866 cases met inclusion criteria, comprising 21,114 colorectal, 31,761 lung, 23,757 female breast, and 15,314 prostate cancers. Between 2001 and 2006, there was no consistent association between socio-economic circumstances and colorectal cancer incidence, but 2006–2012 saw an emerging deprivation gradient in both sexes. The incidence rate ratio (IRR) for colorectal cancer between most deprived and least deprived increased from 1.03 (95% confidence interval [CI] 0.91–1.16) to 1.24 (95% CI 1.11–1.39) during the study period. The incidence of lung cancer showed the strongest relationship with socio-economic circumstances, with inequalities widening across the study period among women from IRR 2.66 (95% CI 2.33–3.05) to 2.91 (95% CI 2.54–3.33) in 2001–03 and 2010–12, respectively. Breast and prostate cancer showed an inverse relationship with socio-economic circumstances, with lower incidence among people living in more deprived areas.

**Conclusion:**

Significant socio-economic inequalities remain in cancer incidence in the West of Scotland, and in some cases are increasing. In particular, this study has identified an emerging, previously unreported, socio-economic gradient in colorectal cancer incidence among women as well as men. Actions to prevent, mitigate, and undo health inequalities should be a public health priority.

## Highlights

• There is an emerging social gradient in colorectal cancer incidence in both sexes.
• Profound socio-economic inequalities in lung cancer persist.
• Breast and prostate cancer remain more common among less deprived populations.

## Introduction

Cancer is the most common cause of death, and of premature death, in Scotland, with four sites, lung, breast, prostate, and colorectal, accounting for approximately half of all cases and deaths.^1^, ^2^, ^3^ Scotland experiences higher rates of cancer incidence and mortality than the rest of the UK, with the burden particularly high in the West of Scotland.^4^

Socio-economic inequalities in incidence have been described for a range of cancers worldwide, across various measures of socio-economic circumstances.^5^ Global trends toward population ageing and a growing burden of noncommunicable disease suggest that in coming years, cancer will become an increasingly important proximal cause of health inequalities. Since exposure to modifiable factors is a key determinant of cancer risk,^6^ studying inequalities in incidence may identify opportunities to improve the reach and effectiveness of health improvement activities.

Although previous studies have documented the existence of such inequalities, there is a lack of up-to-date analyses from high-income countries, particularly in relation to trends over time. Recent years have seen changes in the distribution of risk factors (such as tobacco use), in primary and secondary prevention efforts (such as screening), and in the economic and political forces that drive the social determinants of health.^7^, ^8^, ^9^ There is thus a need to update our understanding of socio-economic inequalities in cancer incidence.

We investigated the relationship between socio-economic circumstances and incidence of the four most common cancers in the West of Scotland between 2001 and 2012, using data from a population-based registry.

## Methods

### Study population

For the purpose of this study, the West of Scotland region was defined as comprising the Health Board areas of Ayrshire and Arran, Dumfries and Galloway, Forth Valley, Greater Glasgow and Clyde, and Lanarkshire. Together, these areas have a resident population of approximately 2.5 million people; around half of the total Scottish population.

Data on cases were obtained from the West of Scotland Cancer Surveillance Unit, which holds regional data from the Scottish Cancer Registry.^10^ Inclusion criteria were incident case of colorectal, lung, prostate, or female breast cancer; aged ≥15 years; date of incidence between 2001 and 2012; resident at diagnosis in any one of the following Health Boards, Ayrshire and Arran, Dumfries and Galloway, Forth Valley, Greater Glasgow and Clyde, and Lanarkshire. Exclusion criteria were inability to ascertain deprivation status due to missing postcode or residence in a postcode with no SIMD score assigned; multiple registrations for cancers of the same site in the same individual (only the earliest registration for each site in each individual was included); cases with a negative survival time or recorded as having a hospital admission after death (assumed to represent linkage errors resulting from probabilistic matching).

The date of incidence for each case was defined in the registry as the first outpatient consultation, hospital admission, pathology report, or treatment for that cancer; or, if none of the aforementioned criteria could be established, as the date of diagnosis or best estimate. Year of incidence was classified into four 3-year periods to facilitate analysis. These were chosen to correspond with an extension of the upper age limit for breast cancer screening (between 2004 and 2006) and the introduction of a national screening programme for colorectal cancer (between 2007 and 2009).

Like most other population-based cancer registries, the Scottish Cancer Registry does not collect individual-level socio-economic indicators, such as income or occupation. An area-level proxy indicator, the Scottish Index of Multiple Deprivation (SIMD), was therefore used, based on the postcode of each case at diagnosis. The SIMD is based on a relative ranking of 6505 small areas (‘datazones’), according to the weighted sum of scores from seven domains (income, employment, crime, education, health, housing and access to amenities and services). Datazones have a mean population of 800 individuals; their boundaries remained stable throughout the study period of 2001–2012. There have been multiple releases of SIMD over the years: for this analysis, SIMD 2006 was chosen as the release closest to the midpoint of the study period. Cases were classified on the basis of population-weighted quintiles of SIMD score, with one representing the least deprived and five the most deprived sectors of the population.

Midyear population estimates, adjusted for the results of the 2011 census, were obtained from National Records for Scotland for each datazone, by age, sex and year.

### Analysis

All analyses were undertaken using Stata, version 12 (Statacorp, College Station, TX).

Crude incidence rates, in cases per 100,000 person-years, were calculated for each 3-year period and for the study period as a whole by dividing the cumulative number of incident cases occurring during that period by the cumulative population for each year of that period.

In order to adjust for the local age profile and enable comparison with published studies from other regions and countries, age-standardisation of incidence rates was undertaken using the direct method, with the European Standard Population (truncated to those aged 15 years and above) as reference.^11^

Poisson regression was used to investigate the relationship between SIMD quintile and incidence of each of the four cancers. Further details of these methods are provided in the online supplementary material.

To determine if there was a linear trend over time in the incidence rate ratio (IRR) between the most and least deprived areas, we used weighted least square regressions of IRR against year. The weights used were inversely proportional to the error variance of the IRRs.

### Ethical considerations

Data included in this study were collected by the Scottish Cancer Registry and held by the West of Scotland Cancer Surveillance Unit for the purposes of service monitoring and quality improvement. The Caldicott guardian of each participating NHS Health Board has given permission for the West of Scotland Cancer Surveillance Unit to hold these data, and the Privacy Advisory Committee of NHS National Services Scotland has previously approved the use of these data for research.

## Results

A total of 93,866 cases meeting the inclusion criteria were identified from the registry.

In total, 1920 cases (2.0%) were excluded from all analyses for the following reasons: multiple registrations for the same site in the same individual (n = 1783); duplicate records of same cancer episode (n = 55); record of hospital admission following recorded date of death (n = 54); negative survival time (n = 18); or no data on deprivation status (n = 10). The number of cases excluded due to missing data on deprivation status was too small to allow meaningful analysis of how they differed from cases for whom these data were available. The study population therefore comprised 91,946 cases, as shown in Supplementary Fig. 1.

During the period 2001 to 2012, there were 21,114 cases of colorectal cancer, 31,761 cases of lung cancer, 23,757 cases of female breast cancer, and 15,314 cases of prostate cancer registered in the West of Scotland. Their characteristics—and those of the population of the West of Scotland, as of 2006—are shown in Table 1.

**Table 1:** Characteristics of cases of colorectal, lung, breast, and prostate cancer (2001–2012), and of the population (2006) for the West of Scotland.

Characteristic	Incident cases by cancer site 2001–2012								Population estimate in 100,000s in 2006	
	Colorectal		Lung		Breast		Prostate		West of Scotland	
	n	%	n	%	n	%	n	%	n	%
Age group (y)										
15–44	506	2.4	325	1.0	2440	10.3	22	0.1	10.4	49.3
45–54	1530	7.3	1692	5.3	4789	20.2	517	3.4	3.6	16.8
55–64	3955	18.7	6033	19.0	5884	24.8	3183	20.8	3.1	14.4
65–74	6681	31.6	11,122	35.0	5170	21.8	6055	39.5	2.3	10.8
75–84	6255	29.6	10,008	31.5	3810	16.0	4,41	28.8	1.4	6.6
85 plus	2187	10.4	2581	8.1	1664	7.0	1124	7.3	0.4	2.1
Sex										
Male	11,428	54.1	16,720	52.6	–	–	15,314	100.0	10.0	47.2
Female	9686	45.9	15,041	47.4	23,757	100.0	–	–	11.2	52.8
SIMD 2006 quintile										
1 (least deprived)	3203	15.2	2654	8.4	4114	17.3	2877	18.8	3.4	15.9
2	3085	14.6	3310	10.4	3856	16.2	2636	17.2	3.3	15.6
3	3762	17.8	4886	15.4	4292	18.1	2781	18.2	3.8	17.9
4	5085	24.1	8061	25.4	5458	23.0	3412	22.3	4.8	22.5
5 (most deprived)	5979	28.3	12,850	40.5	6037	25.4	3608	23.6	6.0	28.1
Total	21,114	100.0	31,761	100.0	23,757	100.0	15,314	100.0	21.2	100.0

### Incidence

**Fig. 1:**
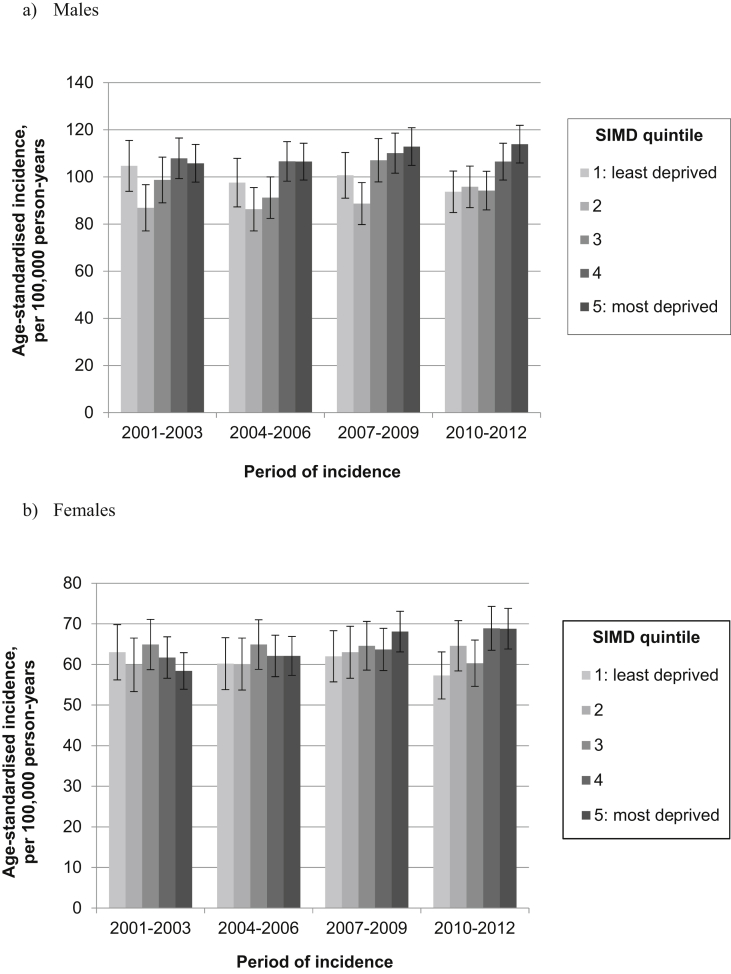
Age-standardised incidence of colorectal cancer for (a) males and (b) females, by deprivation quintile and period of incidence (with 95% confidence intervals).

**Fig. 2:**
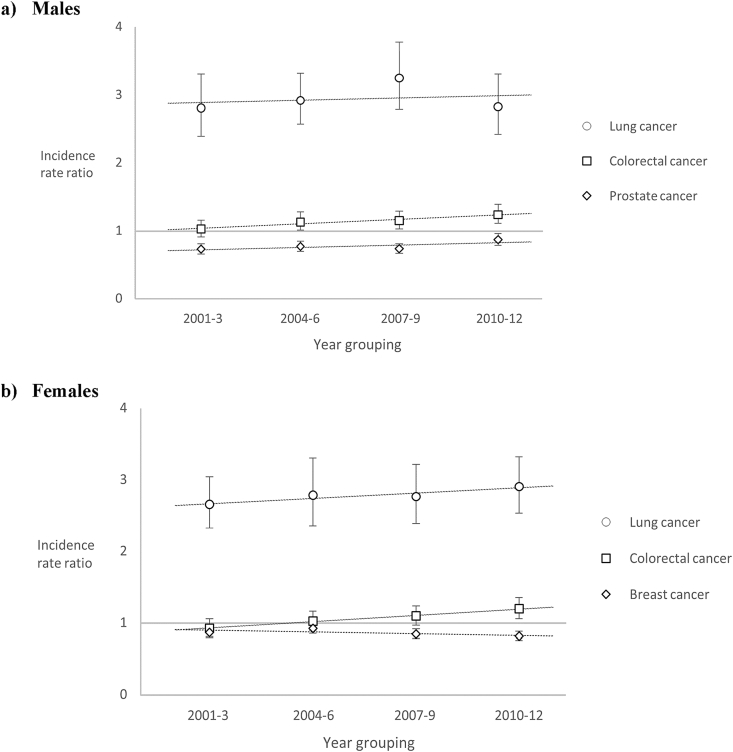
Trends in age-adjusted incidence rate ratio for (a) males and (b) females between most and least deprived quintiles.

**Fig. 3:**
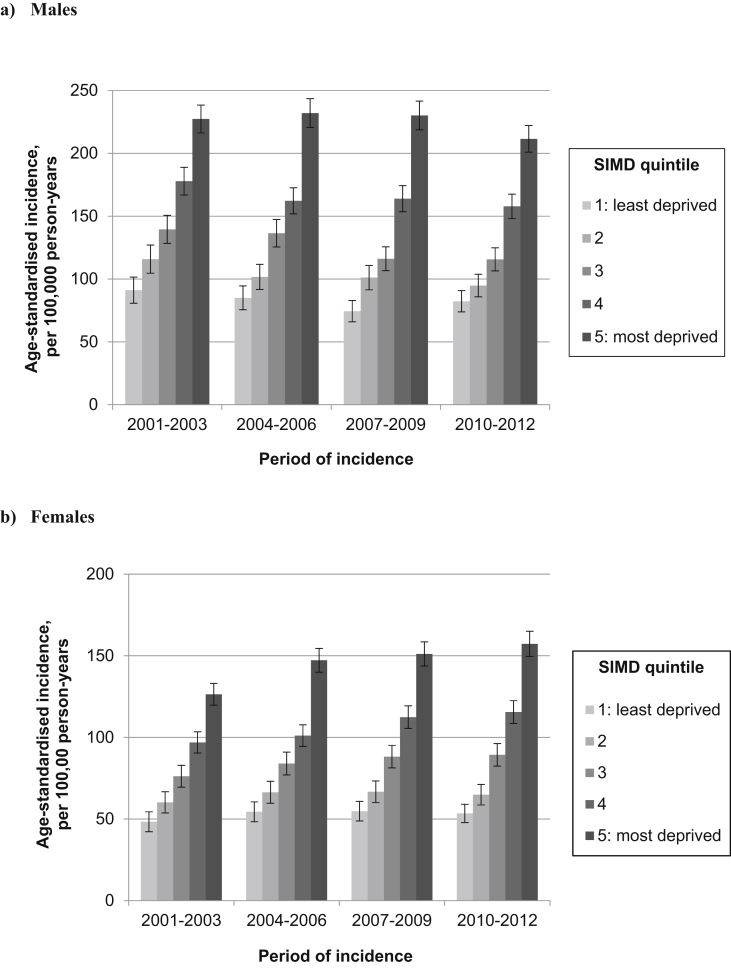
Age-standardised incidence of lung cancer for (a) males and (B) females, by deprivation quintile and period of incidence (with 95% confidence intervals).

**Fig. 4:**
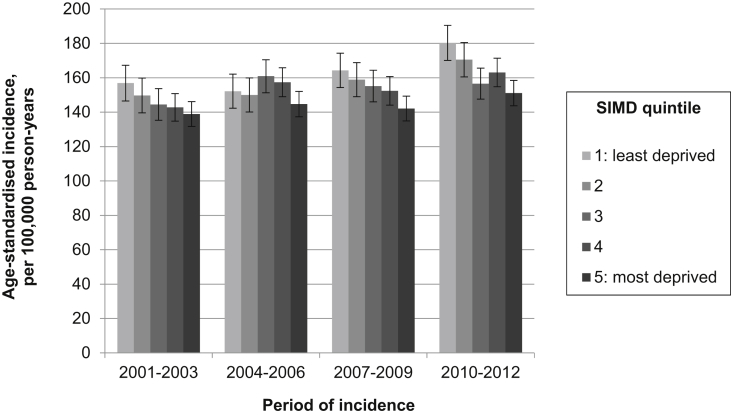
Age-standardised incidence of breast cancer, by deprivation quintile and period of incidence (with 95% confidence intervals).

Fig. 1, Fig. 2, Fig. 3, Fig. 4 illustrate age-standardised incidence rates for each site, by deprivation quintile and period of incidence. Detailed results of incidence analyses are available in the online supplementary material, Supplementary Tables 1–4.

### Colorectal cancer

Among men, the overall age-standardised incidence of colorectal cancer remained stable across the study period (Supplementary Table 1a): in both 2001–2003 and 2010–2012, the rate across all quintiles combined was 101.9 per 100,000 person-years. Incidence tended to be lowest in the second least-deprived quintile and highest in the two most deprived quintiles (Fig. 1a). A socio-economic gradient became more apparent during the latter part of the study period, as a result of declining incidence in the least-deprived quintile and an increase in the most-deprived quintile. The IRR between the most deprived and least-deprived areas therefore increased from 1.03 (95% confidence interval [CI] 0.91–1.16) to 1.24 (95% CI 1.11–1.39) during the study period (Fig. 2a and Supplementary Table 1a).

There was a slight increase in the age-standardised incidence rate of female colorectal cancer during the study period, from 61.1 per 100,000 person-years in 2001–2003 to 68.8 per 100,000 person-years in 2010–2012, though rates among women remained significantly lower than among men (Supplementary Table 1b). Among women, incidence showed less variation by deprivation than among men, with no clear association between socio-economic circumstances and age-standardised incidence of colorectal cancer during the first half of the study period (Fig. 1b). However, the second half saw the emergence of a socio-economic gradient, driven by an increase in incidence in the two most deprived quintiles and, to a lesser extent, a decline in incidence among the least deprived quintile. The IRR between women living in the most and least deprived quintiles therefore increased from 0.93 (95% CI 0.82–1.06) in 2001–2003 to 1.20 (95% CI 1.06–1.36) in 2010–2012 (Fig. 2b and Supplementary Table 1b). This trend was also observed in sensitivity analyses using the 2009 release of SIMD and 4-year rather than 3-year periods (data not shown).

### Lung cancer

The overall age-standardised incidence of lung cancer showed opposing trends by sex (Supplementary Tables 2a and 2b), decreasing over time among men (from 162.4 per 100,000 person-years in 2001–2003 to 139.6 per 100,000 person-years in 2010–2012), but increasing among women (from 89.6 per 100,000 person-years in 2001–2003 to 103.2 per 100,000 person-years in 2010–2012).

Marked inequalities in age-standardised lung cancer incidence were observed in both sexes, with a clear stepwise socio-economic gradient and an almost three-fold difference between those living in the most and least deprived areas (Fig. 3).

Among men, the gradient became more pronounced between 2001–2003 and 2007–2009 as incidence declined among the least deprived (Supplementary Table 2a). The IRR between men living in the most and least deprived quintiles therefore increased from 2.81 (95% CI 2.39–3.31) in 2001–2003 to 3.25 (95% CI 2.79–3.78) in 2007–2009. However, this trend was reversed in the most recent three-year period, with a slight lessening of the gradient and a reduction in the IRR for 2010–2012 to 2.93 (95% CI 2.42–3.31).

In contrast, among women, the IRR between the most and least deprived quintiles grew over time during the study period from 2.66 (95% CI 2.33–3.05) in 2001–2003 to 2.91 (95% CI 2.54–3.33) in 2010–2012 (Fig. 2b and Supplementary Table 2b). This was explained by increasing incidence among the most deprived and static or declining incidence among the less deprived.

### Breast cancer

The age-standardised incidence of breast cancer increased in each successive three-year study period, from 145.3 per 100,000 person-years in 2001–2003 to 162.9 per 100,000 person-years in 2010–2012 (Supplementary Table 3). A clear inverse socio-economic gradient was observed, with incidence negatively associated with deprivation (Fig. 4). Though this inequality appeared to grow over time, with the IRR between the most and least deprived quintiles decreasing from 0.87 (95% CI 0.80–0.95) in 2001–2003 to 0.82 (95% CI 0.76–0.89) in 2010–2012 (Supplementary Table 3); this difference was not statistically significant.

### Prostate cancer

**Fig. 5:**
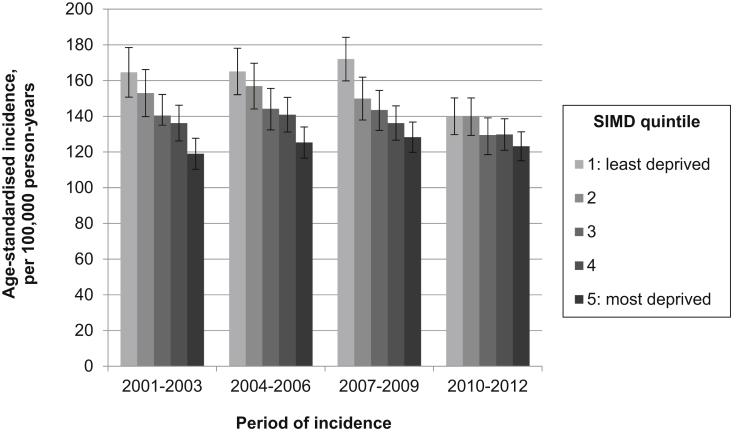
Age-standardised incidence of prostate cancer, by deprivation quintile and period of incidence (with 95% confidence intervals).

No clear trend in the overall age-standardised incidence of prostate cancer was observed across the study period (Supplementary Table 4): for instance, incidence in 2001–2003 was 138.9 per 100,000 person-years and in 2010–2012, 131.6 per 100,000 person-years. During the early years of the study period, incidence tended to be higher among men with more favourable socio-economic circumstances (Fig. 5). However, this socio-economic gradient was noticeably less pronounced in 2010–2012, when a substantial decline in incidence was observed in all quintiles and particularly among men living in the least deprived areas. This was also reflected in a declining socio-economic gap, with the IRR between the most and least deprived quintiles increasing from 0.73 (95% CI 0.66–0.81) in 2001–2003 to 0.87 (95% CI 0.76–0.96) (Fig. 2 and Supplementary Table 4).

### Trends in IRRs

Fig. 2 shows the trends in the IRR between the most and least deprived quintiles for each cancer, by sex. The figure suggests a general increase in the relative differentials over time between the most and least deprived quintiles for each cancer, with the exception of prostate cancer. Only the trend for colorectal cancer was statistically significant (*P* = 0.002 for women and *P* = 0.026 for men).

## Discussion

This study aimed to describe the incidence of the four most common cancers according to socio-economic circumstances over a 12-year period in the West of Scotland. It found that this relationship varied by cancer site and over time.

The finding of a new and increasing socio-economic inequality in colorectal cancer incidence extends the results of a previous study from the West of Scotland which reported an association between deprivation and incidence between 2005 and 2007 in males only.^12^ The magnitude of the deprivation gap observed here is consistent with that study and with others from elsewhere in the UK.^12^, ^13^, ^14^ However, an emerging socio-economic gradient in female colorectal cancer incidence in more recent years has not previously been reported and is of concern. Though the confidence intervals were relatively large, these findings were robust to sensitivity analyses.

Strong evidence exists to link socio-economic disadvantage with exposures known to increase the risk of colorectal cancer, though few studies have explored changes over time in the distribution of these risk factors that might explain the trends observed here.^15^, ^16^, ^17^

The launch of a national bowel screening programme, uptake of which is significantly lower among those from more deprived areas,^18^ might have been expected to attenuate or reverse the emerging socio-economic gradient as a result of the detection of prevalent or indolent cancers among those screened. However, the two least deprived quintiles showed only a slight increase in colorectal cancer incidence during screening rollout (2007–2009), followed by a substantial decline in the least deprived quintile during full implementation (2010–12). Though screening does have a primary preventative role through the removal of pre-cancerous polyps, it is likely too soon to observe this benefit in the population. Further analyses of these data stratified by screening status are required to fully interpret these findings.

The finding of a strong socio-economic gradient in lung cancer incidence in both sexes is in keeping with existing studies, with the observation of an almost three-fold greater incidence in the most compared to the least deprived consistent with previous estimates from Scotland^19^ and elsewhere in the UK.^19^, ^20^ Given that lung cancer is the most common cancer in Scotland, and survival following diagnosis remains poor, this translates to a substantial excess of morbidity and mortality among the most deprived.

The socio-economic gradient in lung cancer incidence largely reflects a socio-economic gradient in smoking, responsible for an estimated 80–85% of lung cancers.^21^ Multiple studies have demonstrated widening socio-economic inequalities in smoking prevalence in the UK over recent decades, which could explain the trends described here.^7^, ^22^, ^23^, ^24^

The finding of a positive association between socio-economic position and breast cancer incidence also coincides with previous studies and can largely be explained by socio-economic differences in reproductive history and screening uptake.^13^, ^14^, ^25^, ^26^ Though the socio-economic gradient in breast cancer incidence was most pronounced in the most recent period, data from the Scottish Breast Screening Programme suggest that changes in screening uptake by deprivation are unlikely to be responsible.^27^ No other recent evidence on trends in the socio-economic distribution of risk factors could be identified, which might help interpret this finding.

The finding of a higher incidence of prostate cancer among men living in less deprived areas is also consistent with previous research.^13^, ^14^ In the absence of known modifiable risk factors, this may reflect higher rates of prostate-specific antigen testing among less deprived men and hence higher rates of overdiagnosis.^28^, ^29^, ^30^ The decline in incidence among the least deprived in the most recent study period is as yet unexplained but may reflect changes in clinical practice. Although ethnicity is associated with prostate cancer risk,^31^ and with socio-economic position,^32^ it is unlikely to be an important confounder of these results given that less than 2% of the catchment population are of non-White ethnicity.^32^

This study's strengths include the use of a population-based cancer registry with high levels of case ascertainment and data completeness.^33^ In particular, the near-complete availability of data on postcode of residence and hence on area-level socio-economic circumstances compares favourably to other registries.^34^, ^35^ Using data from a single national registry also ensured consistency of data collection and coding.

One limitation was our reliance on an area-level rather than individual-level socio-economic indicator, which may have resulted in an underestimation of deprivation gradients in cancer.^36^, ^37^ The SIMD datazone is among the smallest output areas used for this purpose and aims to reflect natural communities where possible; however, the potential for heterogeneity, and therefore bias towards the null, remains. The use of a single SIMD release from the approximate midpoint of the study period was a pragmatic decision to facilitate identification of deprivation-specific population denominators and ensure consistency in the indicators included but might result in misclassification of exposure among cases from each extreme of the study period.

### Conclusion

Significant socio-economic inequalities remain in cancer incidence in the West of Scotland. The stubborn persistence of stark inequalities in lung cancer incidence, and the emergence of a modest socio-economic gradient for colorectal cancer are of particular concern.

There is strong evidence for socio-economic variation in the individual-level risk factors for these conditions, evidencing a need to target health improvement resources and activities in order to achieve ‘proportionate universalism’.^38^ However, such variation begs a wider question as to the ultimate drivers of such behavioural differences and identifies the need to address the ‘fundamental causes’ of health inequality, in the form of the unequal distribution of wealth, resources and power.^39^

Those cancers showing an inverse socio-economic gradient are those less likely to be explained by modifiable risk factors. However, a higher incidence of both breast and prostate cancer among the less deprived may to an extent reflect inequalities in ‘over-diagnosis’ (or ‘over-detection’), with the attendant burdens of unnecessary investigations, treatment, anxiety and healthcare cost.

Future research should focus on how health improvement activities can best be targeted or adapted in order to prevent and mitigate socio-economic inequalities in the incidence of cancer and other potentially avoidable conditions.

## Author statements

### Acknowledgements

The authors thank Billy Sloan for data management and IT support.

### Ethical approval

Data included in this study were collected by the Scottish Cancer Registry and held by the West of Scotland Cancer Surveillance Unit for the purposes of service monitoring and quality improvement. The Caldicott Guardian of each participating NHS Health Board has given permission for the West of Scotland Cancer Surveillance Unit to hold these data and the Privacy Advisory Committee of NHS National Services Scotland has previously approved the use of these data for research.

### Funding

This work was undertaken as part of a Masters in Public Health, funded by NHS Greater Glasgow and Clyde. EJT is currently affiliated to the MRC/CSO Social and Public Health Sciences Unit, funded by the Medical Research Council (MC_UU_12017/13 and MC_UU_12017/15) and Scottish Government Chief Scientist Office (SPHSU13 and SPHSU 15).

### Competing interests

None declared.

## Appendix A Supplementary data

mmc1
