# Supplementary material for: Socio-economic inequalities in the incidence of four common cancers: a population-based registry study
Source: Public Health. 2018 Jan;154:1–10. doi: 10.1016/j.puhe.2017.10.005 (PMC5764071; doi:10.1016/j.puhe.2017.10.005)
Supplement: mmc1 [file mmc1.docx]

**Supplementary Figure 1. Flowchart for study population.**

**
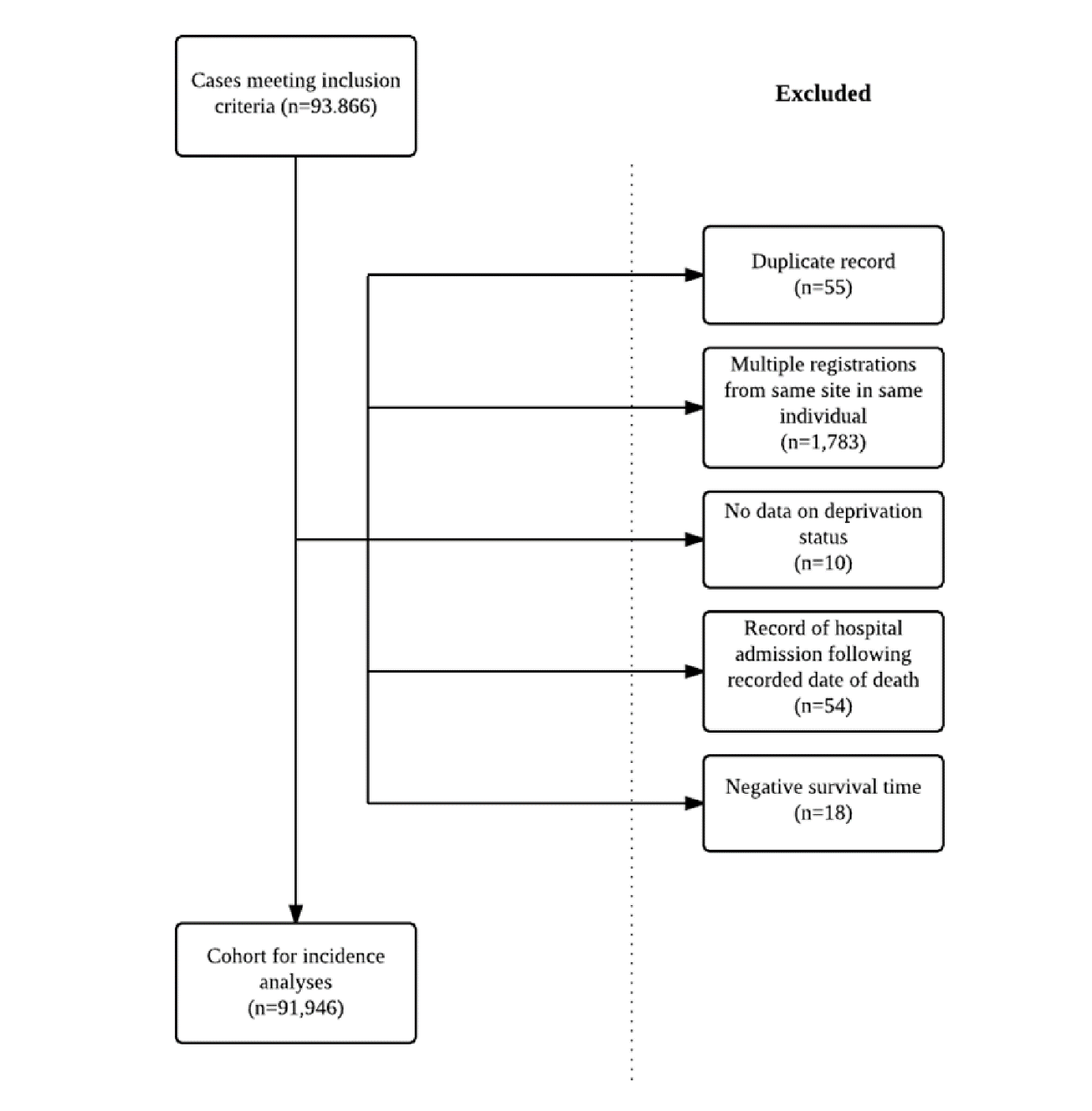
**

**Methods for Poisson regression**

Poisson regression was used to investigate the relationship between SIMD quintile and incidence of each of the four cancers, using Stata version 12 (Statacorp: College Station, TX). Poisson models were stratified by period of incidence and, where relevant, sex, and comprised the independent variables SIMD quintile and age group and the dependent variable number of cases, with person-years as the exposure. The overall significance of individual variables was assessed using a Wald test and the fit of each model was assessed using the goodness of fit chi-squared test. The presence of over-dispersion was checked by constructing negative binomial regression models with the same covariates as the final Poisson model and examining the results of the likelihood ratio test for the over-dispersion parameter. The results suggested significant over-dispersion only in the data for lung cancer: incidence rate ratios and associated 95% confidence intervals for this site were therefore derived by negative binomial rather than Poisson regression.

**Supplementary Table 1a. Crude and age-standardised incidence rates of male colorectal cancer by deprivation quintile and period of incidence.**

|  | Crude incidence  (per 100,000 person-years) | Age-standardised incidence^1^  (per 100,000 person-years) | 95% confidence interval | Absolute rate difference | 95% confidence interval | Adjusted incidence rate ratio^2^ | 95% confidence interval | P value |
| --- | --- | --- | --- | --- | --- | --- | --- | --- |
| 2001-2003 |  |  |  |  |  |  |  |  |
| 1: least deprived | 69.0 | 104.7 | 93.9 – 115.5 | - | - | 1.00 | - | - |
| 2 | 61.0 | 86.9 | 77.1 – 96.8 | -17.8 | -32.5 – -3.1 | 0.84 | 0.73 – 0.97 | 0.019 |
| 3 | 71.7 | 98.7 | 89.0 – 108.3 | -6.0 | -20.4 – 8.4 | 0.96 | 0.84 – 1.10 | 0.540 |
| 4 | 81.5 | 107.9 | 99.3 – 116.6 | 3.2 | -10.7 – 17.1 | 1.05 | 0.93 – 1.19 | 0.432 |
| 5: most deprived | 74.8 | 105.8 | 97.8 – 113.8 | 1.1 | -12.3 – 14.5 | 1.03 | 0.91 – 1.16 | 0.616 |
| All quintiles | 72.6 | 101.9 | 97.8 – 106.0 | - | - | - | - | - |
|  |  |  |  |  |  |  |  |  |
| 2004-2006 |  |  |  |  |  |  |  |  |
| 1: least deprived | 69.4 | 97.6 | 87.3 – 107.8 | - | - | 1.00 | - | - |
| 2 | 64.8 | 86.3 | 77.1 – 95.4 | -11.3 | -25.4 – 2.8 | 0.91 | 0.79 – 1.05 | 0.194 |
| 3 | 69.4 | 91.2 | 82.4 – 100.0 | -6.4 | -20.3 – 7.5 | 0.97 | 0.85 – 1.11 | 0.680 |
| 4 | 82.1 | 106.6 | 98.2 – 115.1 | 9.0 | -4.4 – 22.4 | 1.13 | 1.00 – 1.28 | 0.049 |
| 5: most deprived | 76.1 | 106.5 | 98.7 – 114.4 | 8.9 | -4.5 – 22.3 | 1.13 | 1.01 – 1.28 | 0.038 |
| All quintiles | 73.3 | 99.2 | 95.2 – 103.1 | - | - | - | - | - |
|  |  |  |  |  |  |  |  |  |
| 2007-2009 |  |  |  |  |  |  |  |  |
| 1: least deprived | 79.3 | 100.7 | 91.0 – 110.4 | - | - | 1.00 | - | - |
| 2 | 72.3 | 88.7 | 79.8 – 97.6 | -12.0 | -26.0 – 2.0 | 0.90 | 0.79 – 1.03 | 0.136 |
| 3 | 84.0 | 107.1 | 97.9 – 116.4 | 6.4 | -7.9 – 20.7 | 1.08 | 0.96 – 1.22 | 0.208 |
| 4 | 85.6 | 110.1 | 101.6 – 118.6 | 9.4 | -4.3 – 23.1 | 1.10 | 0.98 – 1.24 | 0.105 |
| 5: most deprived | 80.8 | 112.9 | 104.9 – 120.8 | 12.2 | -1.2 – 25.6 | 1.15 | 1.03 – 1.29 | 0.016 |
| All quintiles | 80.9 | 105.2 | 101.2 – 109.2 | - | - | - | - | - |
|  |  |  |  |  |  |  |  |  |
| 2010-2012 |  |  |  |  |  |  |  |  |
| 1: least deprived | 80.5 | 93.7 | 84.9 – 102.5 | - | - | 1.00 | - | - |
| 2 | 83.4 | 95.8 | 87.0 – 104.5 | 2.1 | -11.8 – 16.0 | 1.05 | 0.93 – 1.19 | 0.454 |
| 3 | 78.5 | 94.2 | 86.0 – 102.4 | 0.5 | -13.1 – 14.1 | 1.04 | 0.92 – 1.18 | 0.494 |
| 4 | 87.4 | 106.5 | 98.7 – 114.4 | 12.8 | -0.6 – 26.2 | 1.19 | 1.06 – 1.33 | 0.003 |
| 5: most deprived | 81.5 | 113.9 | 105.9 – 121.8 | 20.2 | 6.8 – 33.6 | 1.24 | 1.11 – 1.39 | 0.000 |
| All quintiles | 82.4 | 101.9 | 98.2 – 105.6 | - | - | - | - | - |
|  |  |  |  |  |  |  |  |  |

1. European Standard Population 2013

2. Poisson regression: adjusted for age group (0-44; 45-54; 55-64; 65-74; 75-84; ≥85 years).

**Supplementary Table 1b. Crude and age-standardised incidence rates of female colorectal cancer by deprivation quintile and period of incidence.**

|  | Crude incidence  (per 100,000 person-years) | Age-standardised incidence^1^  (per 100,000 person-years) | 95% confidence interval | Absolute rate difference | 95% confidence interval | Adjusted incidence rate ratio^2^ | 95% confidence interval | P value |
| --- | --- | --- | --- | --- | --- | --- | --- | --- |
| 2001-2003 |  |  |  |  |  |  |  |  |
| 1: least deprived | 52.5 | 63.0 | 56.2 – 69.9 | - | - | 1.00 | - | - |
| 2 | 54.2 | 59.9 | 53.3 – 66.4 | -3.1 | -12.6 – 6.4 | 0.95 | 0.81 – 1.11 | 0.504 |
| 3 | 61.9 | 64.9 | 58.7 – 71.1 | 1.9 | -7.4 – 11.2 | 1.04 | 0.90 – 1.20 | 0.612 |
| 4 | 62.4 | 61.7 | 56.6 – 66.9 | -1.3 | -9.9 – 7.3 | 0.98 | 0.86 – 1.12 | 0.777 |
| 5: most deprived | 54.1 | 58.4 | 53.9 – 63.0 | -4.6 | -12.9 – 3.7 | 0.93 | 0.82 – 1.06 | 0.295 |
| All quintiles | 57.1 | 61.1 | 58.6 – 63.6 | - | - | - | - | - |
|  |  |  |  |  |  |  |  |  |
| 2004-2006 |  |  |  |  |  |  |  |  |
| 1: least deprived | 54.2 | 60.2 | 53.8 – 66.6 | - | - | 1.00 | - | - |
| 2 | 56.6 | 60.1 | 53.7 – 66.4 | -0.1 | -9.4 – 9.2 | 1.00 | 0.86 – 1.16 | 0.990 |
| 3 | 62.7 | 64.9 | 58.8 – 71.0 | 4.7 | -4.5 – 13.9 | 1.08 | 0.94 – 1.24 | 0.281 |
| 4 | 62.9 | 62.1 | 57.0 – 67.3 | 1.9 | -6.7 – 10.5 | 1.02 | 0.90 – 1.17 | 0.722 |
| 5: most deprived | 57.1 | 62.1 | 57.3 – 66.8 | 1.9 | -6.4 – 10.2 | 1.03 | 0.90 – 1.17 | 0.647 |
| All quintiles | 58.9 | 62.1 | 59.6 – 64.6 | - | - | - | - | - |
|  |  |  |  |  |  |  |  |  |
| 2007-2009 |  |  |  |  |  |  |  |  |
| 1: least deprived | 59.3 | 62.0 | 55.7 – 68.3 | - | - | 1.00 | - | - |
| 2 | 61.4 | 63.0 | 56.6 – 69.3 | 1.0 | -8.3 – 10.3 | 1.02 | 0.88 – 1.18 | 0.791 |
| 3 | 62.6 | 64.6 | 58.6 – 70.6 | 2.6 | -6.5 – 11.7 | 1.04 | 0.91 – 1.19 | 0.574 |
| 4 | 63.8 | 63.7 | 58.5 – 68.9 | 1.7 | -6.9 – 10.3 | 1.03 | 0.90 – 1.17 | 0.700 |
| 5: most deprived | 61.6 | 68.1 | 63.1 – 73.2 | 6.1 | -2.5 – 14.7 | 1.10 | 0.97 – 1.24 | 0.143 |
| All quintiles | 61.9 | 64.5 | 62.0 – 67.1 | - | - | - | - | - |
|  |  |  |  |  |  |  |  |  |
| 2010-2012 |  |  |  |  |  |  |  |  |
| 1: least deprived | 58.9 | 57.3 | 51.5 – 63.2 | - | - | 1.00 | - | - |
| 2 | 65.9 | 64.6 | 58.4 – 70.9 | 7.3 | -2.0 – 16.6 | 1.12 | 0.97 – 1. 29 | 0.117 |
| 3 | 60.0 | 60.3 | 54.6 – 66.0 | 3.0 | -5.9 – 11.9 | 1.05 | 0.91 – 1.20 | 0.512 |
| 4 | 69.4 | 68.9 | 63.5 – 74.3 | 8.6 | 2.8 – 20.4 | 1.19 | 1.05 – 1.36 | 0.007 |
| 5: most deprived | 61.9 | 68.8 | 63.8 – 73.9 | 8.5 | 2.9 – 20.1 | 1.20 | 1.06 – 1.36 | 0.005 |
| All quintiles | 63.4 | 64.7 | 62.1 – 67.2 | - | - | - | - | - |
|  |  |  |  |  |  |  |  |  |

1. European Standard Population 2013

2. Poisson regression: adjusted for age group (0-44; 45-54; 55-64; 65-74; 75-84; ≥85 years).

**Supplementary Table 2a. Crude and age-standardised incidence rates of male lung cancer, by deprivation quintile and period of incidence.**

|  | Crude incidence  (per 100,000 person-years) | Age-standardised incidence^1^  (per 100,000 person-years) | 95% confidence interval | Absolute rate difference | 95% confidence interval | Adjusted incidence rate ratio^2^ | 95% confidence interval | P value |
| --- | --- | --- | --- | --- | --- | --- | --- | --- |
| 2001-2003 |  |  |  |  |  |  |  |  |
| 1: least deprived | 56.1 | 91.1 | 80.7 – 101.5 | - | - | 1.00 | - | - |
| 2 | 79.8 | 115.8 | 104.6 – 127.0 | 24.7 | 9.4 – 40.0 | 1.35 | 1.13 – 1.62 | 0.001 |
| 3 | 101.2 | 139.5 | 128.3 – 150.8 | 48.4 | 33.0 – 63.8 | 1.63 | 1.37 – 1.94 | <0.001 |
| 4 | 135.6 | 177.8 | 166.8 – 188.8 | 86.7 | 71.6 – 101.8 | 2.11 | 1.78 – 2.49 | <0.001 |
| 5: most deprived | 167.5 | 227.3 | 216.2 – 238.5 | 136.2 | 120.9 – 151.5 | 2.81 | 2.39 – 3.31 | <0.001 |
| All quintiles | 116.9 | 162.4 | 157.3 – 167.5 | - | - | - | - | - |
|  |  |  |  |  |  |  |  |  |
| 2004-2006 |  |  |  |  |  |  |  |  |
| 1: least deprived | 58.5 | 85.0 | 75.5 – 94.6 | - | - | 1.00 | - | - |
| 2 | 75.1 | 101.7 | 91.7 – 111.6 | 16.7 | 2.3 – 31.1 | 1.25 | 1.07 – 1.45 | 0.004 |
| 3 | 101.7 | 136.4 | 125.5 – 147.4 | 51.4 | 36.3 – 66.5 | 1.68 | 1.46 – 1.94 | <0.001 |
| 4 | 125.0 | 162.2 | 151.8 – 172.5 | 77.2 | 62.6 – 91.8 | 2.02 | 1.76 – 2.31 | <0.001 |
| 5: most deprived | 165.9 | 232.0 | 220.5 – 243.6 | 147.0 | 131.4 – 162.6 | 2.92 | 2.57 – 3.32 | <0.001 |
| All quintiles | 113.7 | 154.4 | 149.5 – 159.3 | - | - | - | - | - |
|  |  |  |  |  |  |  |  |  |
| 2007-2009 |  |  |  |  |  |  |  |  |
| 1: least deprived | 56.7 | 74.4 | 65.9 – 82.8 | - | - | 1.00 | - | - |
| 2 | 78.8 | 101.1 | 91.4 – 110.7 | 26.7 | 12.5 – 40.9 | 1.34 | 1.12 – 1.60 | 0.001 |
| 3 | 91.8 | 116.1 | 106.6 – 125.6 | 41.7 | 27.6 – 55.8 | 1.60 | 1.35 – 1.90 | <0.001 |
| 4 | 126.4 | 163.9 | 153.5 – 174.3 | 89.5 | 74.8 – 104.2 | 2.20 | 1.86 – 2.58 | <0.001 |
| 5: most deprived | 163.6 | 230.1 | 218.7 – 241.5 | 155.7 | 140.3 – 171.1 | 3.25 | 2.79 – 3.78 | <0.001 |
| All quintiles | 111.9 | 147.0 | 142.3 – 151.7 | - | - | - | - | - |
|  |  |  |  |  |  |  |  |  |
| 2010-2012 |  |  |  |  |  |  |  |  |
| 1: least deprived | 67.3 | 82.3 | 73.8 – 90.8 | - | - | 1.00 | - | - |
| 2 | 78.3 | 94.8 | 85.8 – 103.7 | 12.5 | -1.2 – 26.2 | 1.19 | 1.00 – 1.42 | 0.056 |
| 3 | 93.3 | 115.6 | 106.4 – 124.7 | 33.3 | 19.5 – 47.1 | 1.48 | 1.25 – 1.75 | <0.001 |
| 4 | 125.3 | 157.8 | 148.1 – 167.5 | 75.5 | 61.3 – 89.7 | 2.07 | 1.76 – 2.43 | <0.001 |
| 5: most deprived | 151.8 | 211.5 | 200.9 – 222.1 | 129.2 | 114.4 – 144.0 | 2.83 | 2.42 – 3.31 | <0.001 |
| All quintiles | 110.4 | 139.6 | 135.2 – 144.0 | - | - | - | - | - |
|  |  |  |  |  |  |  |  |  |

1. European Standard Population 2013

2. Negative binomial regression: adjusted for age group (0-44; 45-54; 55-64; 65-74; 75-84; ≥85 years).

**Supplementary Table 2b. Crude and age-standardised incidence rates of female lung cancer, by deprivation quintile and period of incidence.**

|  | Crude incidence  (per 100,000 person-years) | Age-standardised incidence^1^  (per 100,000 person-years) | 95% confidence interval | Absolute rate difference | 95% confidence interval | Adjusted incidence rate ratio^2^ | 95% confidence interval | P value |
| --- | --- | --- | --- | --- | --- | --- | --- | --- |
| 2001-2003 |  |  |  |  |  |  |  |  |
| 1: least deprived | 39.6 | 48.3 | 42.2 – 54.3 | - | – | 1.00 | - | - |
| 2 | 54.3 | 60.2 | 53.7 – 66.8 | 11.9 | 3.0 – 20.8 | 1.27 | 1.08 – 1.50 | 0.004 |
| 3 | 72.5 | 76.2 | 69.5 – 82.9 | 27.9 | 18.9 – 36.9 | 1.61 | 1.38 – 1.88 | <0.001 |
| 4 | 97.4 | 96.9 | 90.4 – 103.3 | 48.6 | 39.8 – 57.4 | 2.03 | 1.77 – 2.34 | <0.001 |
| 5: most deprived | 117.2 | 126.4 | 119.7 – 133.1 | 78.1 | 69.1 – 87.1 | 2.66 | 2.33 – 3.05 | <0.001 |
| All quintiles | 83.3 | 89.6 | 86.6 – 92.7 | - | – | - | - | - |
|  |  |  |  |  |  |  |  |  |
| 2004-2006 |  |  |  |  |  |  |  |  |
| 1: least deprived | 48.4 | 54.4 | 48.3 – 60.6 | - | – | 1.00 | - | - |
| 2 | 61.8 | 66.4 | 59.7 – 73.1 | 12.0 | 3.0 – 21.0 | 1.22 | 1.01 – 1.48 | 0.040 |
| 3 | 80.1 | 84.0 | 77.0 – 90.9 | 30.0 | 20.5 – 38.7 | 1.52 | 1.27 – 1.83 | <0.001 |
| 4 | 102.7 | 101.1 | 94.5 – 107.7 | 46.7 | 37.8 – 55.6 | 1.89 | 1.59 – 2.25 | <0.001 |
| 5: most deprived | 133.8 | 147.2 | 139.9 – 154.6 | 92.8 | 83.3 – 102.3 | 2.79 | 2.36 – 3.31 | <0.001 |
| All quintiles | 92.9 | 98.5 | 95.3 – 101.7 | - | - | - | - | - |
|  |  |  |  |  |  |  |  |  |
| 2007-2009 |  |  |  |  |  |  |  |  |
| 1: least deprived | 51.8 | 54.8 | 48.8 – 60.7 | - | - | 1.00 | - | - |
| 2 | 64.0 | 66.7 | 60.1 – 73.3 | 11.9 | 3.0 – 20.8 | 1.20 | 1.00 – 1.43 | 0.044 |
| 3 | 85.9 | 88.2 | 81.3 – 95.2 | 33.4 | 24.2 – 42.6 | 1.62 | 1.38 – 1.91 | <0.001 |
| 4 | 113.2 | 112.4 | 105.5 – 119.4 | 57.6 | 48.4 – 66.8 | 2.05 | 1.75 – 2.39 | <0.001 |
| 5: most deprived | 136.0 | 151.1 | 143.7 – 158.6 | 96.3 | 86.7 – 105.9 | 2.77 | 2.39 – 3.22 | <0.001 |
| All quintiles | 97.7 | 102.2 | 99.0 – 105.4 | - | - | - | - | - |
|  |  |  |  |  |  |  |  |  |
| 2010-2012 |  |  |  |  |  |  |  |  |
| 1: least deprived | 54.4 | 53.4 | 47.8 – 59.1 | - | - | 1.00 | - | - |
| 2 | 66.3 | 64.9 | 58.6 – 71.2 | 11.5 | 2.8 – 20.2 | 1.22 | 1.05 – 1.42 | 0.011 |
| 3 | 88.7 | 89.3 | 82.4 – 96.2 | 35.9 | 26.8 – 45.0 | 1.66 | 1.43 – 1.93 | <0.001 |
| 4 | 115.9 | 115.5 | 108.5 – 122.5 | 62.1 | 52.9 – 71.3 | 2.14 | 1.87 – 2.46 | <0.001 |
| 5: most deprived | 139.8 | 157.3 | 149.6 – 165.0 | 103.9 | 94.1 – 113.7 | 2.91 | 2.54 – 3.33 | <0.001 |
| All quintiles | 100.6 | 103.2 | 100.0 – 106.4 | - | - | - | - | - |

1. European Standard Population 2013

2. Negative binomial regression: adjusted for age group (0-44; 45-54; 55-64; 65-74; 75-84; ≥85 years).

**Supplementary Table 3. Crude and age-standardised incidence rates of female breast cancer, by deprivation quintile and period of incidence.**

|  | Crude incidence  (per 100,000 person-years) | Age-standardised incidence^1^  (per 100,000 person-years) | 95% confidence interval | Absolute rate difference | 95% confidence interval | Adjusted incidence rate ratio^2^ | 95% confidence interval | P value |
| --- | --- | --- | --- | --- | --- | --- | --- | --- |
|  |  |  |  |  |  |  |  |  |
| 2001-2003 |  |  |  |  |  |  |  |  |
| 1: least deprived | 144.3 | 156.9 | 146.5 – 167.2 | - | - | 1.00 | - | - |
| 2 | 141.6 | 149.7 | 139.6 – 159.8 | -7.2 | -21.6 – 7.2 | 0.96 | 0.87 - 1.05 | 0.347 |
| 3 | 138.8 | 144.5 | 135.3 – 153.7 | -12.4 | -26.2 – 1.4 | 0.92 | 0.84 – 1.01 | 0.078 |
| 4 | 138.7 | 142.8 | 134.8 – 150.8 | -14.1 | -27.1 – -1.1 | 0.91 | 0.83 – 0.99 | 0.023 |
| 5: most deprived | 125.1 | 138.9 | 131.7 – 146.0 | -18.0 | -30.5 – -5.5 | 0.87 | 0.80 – 0.95 | 0.001 |
| All quintiles | 136.0 | 145.3 | 141.4 – 149.1 | - | - | - | - | - |
|  |  |  |  |  |  |  |  |  |
| 2004-2006 |  |  |  |  |  |  |  |  |
| 1: least deprived | 147.6 | 152.2 | 142.3 – 162.0 | - | - | 1.00 | - | - |
| 2 | 145.3 | 150.0 | 140.1 – 160.0 | -2.2 | -16.6 – 12.2 | 0.98 | 0.89 – 1.07 | 0.622 |
| 3 | 156.4 | 160.9 | 151.3 – 170.4 | 8.7 | -5.3 – 22.7 | 1.05 | 0.96 – 1.14 | 0.292 |
| 4 | 154.6 | 157.4 | 149.0 – 165.7 | 5.2 | -8.0 – 18.4 | 1.02 | 0.94 – 1.11 | 0.634 |
| 5: most deprived | 131.0 | 144.7 | 137.3 – 152.0 | -7.5 | -20.1 – 5.1 | 0.93 | 0.86 – 1.01 | 0.086 |
| All quintiles | 145.6 | 152.6 | 148.7 – 156.5 | - | - | - | - | - |
|  |  |  |  |  |  |  |  |  |
| 2007-2009 |  |  |  |  |  |  |  |  |
| 1: least deprived | 165.6 | 164.3 | 154.3 - 174.4 | - | - | 1.00 | - | - |
| 2 | 160.7 | 158.9 | 149.0 – 168.9 | -5.4 | -19.8 – 9.0 | 0.98 | 0.90 – 1.07 | 0.600 |
| 3 | 153.7 | 155.2 | 146.0 – 164.4 | -9.1 | -22.9 – 4.7 | 0.95 | 0.87 – 1.03 | 0.241 |
| 4 | 150.2 | 152.4 | 144.1 – 160.4 | -11.9 | -24.9 – 1.1 | 0.93 | 0.86 – 1.01 | 0.069 |
| 5: most deprived | 127.9 | 142.1 | 134.9 – 149.4 | -21.3 | -34.8 – -9.6 | 0.85 | 0.79 – 0.92 | <0.001 |
| All quintiles | 148.5 | 152.9 | 149.0 – 156.7 | - | - | - | - | - |
|  |  |  |  |  |  |  |  |  |
| 2010-2012 |  |  |  |  |  |  |  |  |
| 1: least deprived | 192.4 | 180.3 | 170.1- 190.4 | - | - | 1.00 | - | - |
| 2 | 179.6 | 170.5 | 160.5 – 180.4 | -9.8 | -24.1 – 4.5 | 0.95 | 0.88 – 1.03 | 0.205 |
| 3 | 157.8 | 156.6 | 147.6 – 165.7 | -23.7 | -37.4 – -10.0 | 0.87 | 0.80 – 0.94 | <0.001 |
| 4 | 161.9 | 163.1 | 154.8 – 171.5 | -17.2 | -30.5 – -3.9 | 0.90 | 0.83 – 0.97 | 0.006 |
| 5: most deprived | 136.4 | 151.1 | 143.7 – 158.7 | -29.2 | -42.0 – -16.4 | 0.82 | 0.76 – 0.89 | <0.001 |
| All quintiles | 161.5 | 162.9 | 159.0 – 166.9 | - | - | - | - | - |

1. European Standard Population 2013

2. Poisson regression: adjusted for age group (0-44; 45-54; 55-64; 65-74; 75-84; ≥85 years).

**Supplementary Table 4. Crude and age-standardised incidence rates of prostate cancer, by deprivation quintile and period of incidence.**

|  | Crude incidence  (per 100,000 person-years) | Age-standardised incidence^1^  (per 100,000 person-years) | 95% confidence interval | Absolute rate difference | 95% confidence interval | Adjusted incidence rate ratio^2^ | 95% confidence interval | P value |
| --- | --- | --- | --- | --- | --- | --- | --- | --- |
|  |  |  |  |  |  |  |  |  |
| 2001-2003 |  |  |  |  |  |  |  |  |
| 1: least deprived | 101.8 | 164.6 | 150.7 – 178.5 | - | - | 1.00 | - | - |
| 2 | 102.9 | 153.0 | 139.8 – 166.2 | -11.6 | -30.8 – 7.6 | 0.95 | 0.85 – 1.06 | 0.367 |
| 3 | 96.7 | 140.4 | 128.6 – 152.2 | -24.2 | -42.4 – -6.0 | 0.85 | 0.76 – 0.96 | 0.006 |
| 4 | 99.5 | 136.2 | 126.2 – 146.2 | -28.4 | -45.5 – -11.3 | 0.84 | 0.75 – 0.93 | 0.001 |
| 5: most deprived | 80.7 | 119.0 | 110.3 – 127.7 | -45.6 | -62.0 – -29.2 | 0.73 | 0.66 – 0.81 | <0.001 |
| All quintiles | 94.6 | 138.9 | 134.0 – 143.9 | - | - | - | - | - |
|  |  |  |  |  |  |  |  |  |
| 2004-2006 |  |  |  |  |  |  |  |  |
| 1: least deprived | 116.0 | 165.1 | 152.1 – 178.2 | - | - | 1.00 | - | - |
| 2 | 113.5 | 156.9 | 144.1 – 169.7 | -8.2 | -27.1 – 10.7 | 0.95 | 0.85 – 1.06 | 0.353 |
| 3 | 106.0 | 144.2 | 132.8 – 155.6 | -20.9 | -38.9 – -2.9 | 0.88 | 0.79 – 0.98 | 0.017 |
| 4 | 107.6 | 140.9 | 131.2 – 150.7 | -24.2 | -41.2 – -7.2 | 0.87 | 0.79 – 0.97 | 0.009 |
| 5: most deprived | 87.1 | 125.3 | 116.6 – 133.9 | -39.8 | -56.1 – -23.5 | 0.77 | 0.70 – 0.85 | <0.001 |
| All quintiles | 103.8 | 143.5 | 138.7 – 148.3 | - | - | - | - | - |
|  |  |  |  |  |  |  |  |  |
| 2007-2009 |  |  |  |  |  |  |  |  |
| 1: least deprived | 138.1 | 172.0 | 159.8 – 184.3 | - | - | 1.00 | - | - |
| 2 | 119.0 | 149.9 | 137.9 – 161.9 | -22.1 | -40.5 – -3.7 | 0.85 | 0.77 - 0.94 | 0.002 |
| 3 | 109.7 | 143.5 | 132.5 – 154.4 | -28.5 | -46.2 – -10.8 | 0.81 | 0.73 - 0.89 | <0.001 |
| 4 | 104.4 | 136.2 | 126.6 – 145.7 | -35.8 | -52.6 – -19.0 | 0.77 | 0.70 - 0.85 | <0.001 |
| 5: most deprived | 90.7 | 128.3 | 119.8 – 136.8 | -43.7 | -60.0 – -27.4 | 0.74 | 0.67 – 0.81 | <0.001 |
| All quintiles | 109.2 | 143.7 | 139.1 – 148.3 | - | - | - | - | - |
|  |  |  |  |  |  |  |  |  |
| 2010-2012 |  |  |  |  |  |  |  |  |
| 1: least deprived | 126.0 | 140.0 | 129.7 – 150.4 | - | - | 1.00 | - | - |
| 2 | 122.4 | 139.8 | 129.3 – 150.4 | -0.2 | -17.7 – 17.3 | 0.98 | 0.89 – 1.09 | 0.761 |
| 3 | 106.9 | 129.5 | 119.9 – 139.1 | -10.5 | -27.4 – 6.4 | 0.91 | 0.82 – 1.01 | 0.075 |
| 4 | 105.4 | 129.8 | 121.0 – 138.5 | -10.2 | -26.6 – 6.2 | 0.92 | 0.84 – 1.02 | 0.098 |
| 5: most deprived | 88.6 | 123.2 | 115.1 – 131.3 | -16.8 | -32.9 – -0.7 | 0.87 | 0.79 – 0.96 | 0.05 |
| All quintiles | 106.9 | 131.6 | 127.4 – 135.7 | - | - | - | - | - |

1. European Standard Population 2013
2. Poisson regression: adjusted for age group (0-44; 45-54; 55-64; 65-74; 75-84; ≥85 years).
